# Validating estimates of problematic drug use in England

**DOI:** 10.1186/1471-2458-7-286

**Published:** 2007-10-10

**Authors:** Martin Frisher, Heath Heatlie, Matthew Hickman

**Affiliations:** 1 Department of Medicines Management, School of Pharmacy, Keele University, ST5 5BG, UK; 2 Department of Social Medicine, University of Bristol, Bristol, BS8 2PR, UK

## Abstract

**Background:**

UK Government expenditure on combatting drug abuse is based on estimates of illicit drug users, yet the validity of these estimates is unknown. This study aims to assess the face validity of problematic drug use (PDU) and injecting drug use (IDU) estimates for all English Drug Action Teams (DATs) in 2001. The estimates were derived from a statistical model using the Multiple Indicator Method (MIM).

**Methods:**

Questionnaire study, in which the 149 English Drug Action Teams were asked to evaluate the MIM estimates for their DAT.

**Results:**

The response rate was 60% and there were no indications of selection bias. Of responding DATs, 64% thought the PDU estimates were about right or did not dispute them, while 27% had estimates that were too low and 9% were too high. The figures for the IDU estimates were 52% (about right), 44% (too low) and 3% (too high).

**Conclusion:**

This is the first UK study to determine the validity estimates of problematic and injecting drug misuse. The results of this paper highlight the need to consider criterion and face validity when evaluating estimates of the number of drug users.

## Background

The UK Government's direct annual expenditure on combating illicit drug use is £1.48 billion [1] (2005/2006). By 2007/08, expenditure on treatment will increase to £478 million from £253 million in 2004 [2]. By 2008, the Government's target is to have the capacity to treat 200,000 drug users [3]. However, direct methods of ascertaining the number of problematic drug users in the country are unlikely to yield accurate figures. This is because of multiple response bias affecting the likelihood of problem drug users a) living in a household that would be included in a sample survey, b) agreeing to participate in a sample survey and c) reporting recent problem drug use [4,5]. One alternative method which has been recommended for country wide prevalence estimates is the Multiple Indicator Method (MIM) [6]. This method combines information on prevalence that is available only in a few areas (the 'anchor points') and drug indicators (e.g. number of crimes, seizures) that are available in all areas [7]. We previously reported a MIM analysis for all Drug Action Team (DAT) areas in England for 2001 [8]. The MIM involved a three stage process: a) factor analysis of the drug indicators, b) regression analysis linking factor scores to "known" prevalence estimates and c) imputation of estimates in all other DATs. The "known" estimates are obtained by a technique known as capture-recapture. The capture-recapture method (CRM) involves 'capturing' a random sample who are then 'marked' and returned to their habitat [9]. Subsequently, a second random sample are 'recaptured' and the number of marked animals from the first sample are observed. The ratio of marked animals to the recaptured sample size is assumed to be the same as the ratio of the first captured sample to the total population. Thus, if a 'capture' sample of 200 animals are marked and released and a 'recapture' sample of 100 contains 10 animals which are marked, the estimate for the total population would be 2,000 (i.e. 10:100 = 200:2,000). In drug use studies the samples are lists of drug users whose identifiers are recorded by agencies such as treatment centres or the police. More sophisticated versions involving multiple samples have also been developed in order to overcome the stringent assumptions which are required when only two-samples are used [10].

In the MIM study, the regression model explained 32% of the variance for the problematic anchor points and 70% for the injecting anchor points. The median estimates were 287,670 problematic drug users and 93,185 injecting drug users in England in 2001. The definition of problematic drug use was 'current use of illicit opiates, crack-cocaine or benzodiazepines'. The definition of injecting drug use was 'current use of any illicit drug where injecting was the method of drug administration'. These definitions are based on the data collection procedures of those organizations whose data is used in capture-recapture studies. These organizations consist of drug treatment agencies, the police, HIV-test registers and needle and syringe exchanges. As most of these agencies categorise drug users simply as being current users at time of contact, it is not possible to give a more precise timeframe.

### Validity of drug user estimates

As the MIM estimates are based on a statistical model it is important to determine the validity of the results. In other words, does the model correspond to how many problematic and injecting drug users there actually are in each DAT? The issue may be addressed in two ways. Firstly, by comparing the MIM estimates to other prevalence estimation techniques. This is known as criterion-related validity, which may be defined as "comparing values with one or more external criteria, known or believed to measure the attribute under study" [11]. The second method is to ask experts in each of the DATs to evaluate the MIM estimates. This is referred to as face validity, which is the degree to which something appears to measure what it is supposed to measure. While in statistical terms, this is a weaker form of validity, in practical terms it is important to practitioners in the field. In 1993, Reuter argued that prevalence estimates in the United States were not widely used because of policy-makers' perception of their limited credibility [12]. However, the situation has changed over the last decade with the introduction of more scientific approaches to the simulation of hidden population [13]. In the UK prevalence estimates are increasingly used in the policy process, for example in relation to the introduction and placement of syringe exchange programmes [14] and modelling HIV prevalence [15]. The Government's Updated Drug Strategy 2002 explicitly aims to increase the proportion of the "estimated 250,000 problematic drug users" who receive treatment by 2008 [3].

With regard to criterion-related validity, we have previously estimated the number of drug users in England, Wales and Scotland in 1996 using a variety of methods corresponding to different forms of drug use [16]. Between 1996 and 2001, the MIM estimate for England increased by 27.6% while a range of drug indicators increased by an average of 18%. This suggests that the 2001 MIM estimates have acceptable criterion-related validity.

To the best of our knowledge there has only been one previous study into face validity of drug user estimates. Friedman et al [17] estimated the number of injecting drug users for 96 metropolitan areas in the USA in 1996 using a variety of multiplier methods similar to those described above. They assessed validity by asking 143 experts to comment on studies or estimates available for their areas. Of the valid responses, 81% endorsed the estimates, 8% though the estimates were too low and 11% thought they were too high. These results suggest that the indicator methods used in the American study had a high degree of construct and/or face validity.

The aim of the current study is to determine the face validity of the 2001 MIM estimates of problematic drug use (PDU) and injecting drug use (IDU) for the 149 DATS in England.

## Methods

All DATs in England were sent a letter from the Home Office informing them of this study and that all DATs were being asked to respond to a questionnaire. Each DAT was provided with information specific to their area and told that the aim of the exercise was to gain a broad understanding of how DATs view the PDU and IDU estimates. The questionnaire was kept short, so as to ensure as high a response rate as possible.

The questionnaire, in Adobe PDF format was emailed to each DAT by the Home Office via the Regional Managers on 21^st ^June 2004. It was requested that the questionnaire be completed by the person (or persons) best placed to assess the number of drug users in the DAT. The return date for the questionnaire was 9^th ^July 2004; non-responders were followed up at the end of July.

## Results

### Response rate

90 of the 149 DATs returned the form by 29^th ^July 2004, giving a response rate of 60.4%. Responders and non-responders did not differ significantly in terms of their mean DAT populations (320,674 vs. 313,707, t = 0.17, p = 0.86). Responders did not significantly differ from non-responders in terms of their estimated PDU rate (PDU 665 vs. 621 per 100,000 population, x^2 ^= 1.51, p = 0.21).

### Evaluation of PDU and IDU estimates

The first question asked was "In your view, is this estimated number of problematic users for your DAT in 2001 A) about right, B) too low, C) too high, D) don't know, E) other (specify) [Note: we did not provide definitions of "about right", "too low" or "too high". For this and subsequent questions, the percentages refer to responding DATs.]

### PDU estimates

Table 1 shows the DATs' evaluation of the MIM PDU estimates. 76% of responding DATs had an opinion of the PDU estimates. Around one third of DATs thought the estimate was about right, while another third thought they were too low. In the light of this finding, we investigated whether DAT perceptions were related to the estimated PDU rate. There was little variation between the groups of DATs (F = 0.23, p = 0.86). There is no indication that DATs who said their estimate was too low came from especially high prevalence areas.

**Table 1:** Drug Action Teams' evaluation of Problematic Drug User (PDU) estimates

	Frequency	Percent	Valid Percent	Percent excluding "don't know"	Mean PDU Estimate per 100,000 population
1.00 about right	30	20	33	44	661
2.00 too low	29	19	32	43	676
3.00 too high	9	6	10	13	610
4.00 don't know	22	15	24		679
Total	90	60	100	100	665
Missing	59	40			
Total	149	100			

### IDU estimates

The second question asked "In your view, is this estimated number of injecting drug users for your DAT in 2001 – A) about right, B) too low, C) too high, D) don't know, E) other (specify). Table 2 shows how DATs evaluated their MIM IDU estimates. Among responding DATs, 73% expressed an opinion about their DAT estimate. 23% of DATs thought the IDU estimates were "about right". Compared to PDU evaluation, more DATs thought that the IDU estimate was too low (46% vs. 32%). There were no significant differences in terms of DATs' IDU prevalence rate (F = 0.78, p = 0.50, n.s.)

**Table 2:** Drug Action Teams' evaluation of Injecting Drug User (IDU) estimates

	Frequency	Percent	Valid Percent	Percent excluding "don't know"	Mean IDU Estimate per 100,000 population
1.00 about right	21	14	23	32	306
2.00 too low	41	28	46	62	234
3.00 too high	4	3	4	6	300
4.00 don't know	24	16	27		220
Total	90	60	100	100	250
Missing	59	40			
Total	149	100			

### Further analysis of Problematic Drug Use (PDU)

Of the 60 DATs who said their PDU estimates were either too low, too high or don't know, 33 (55%) provided some free text information to clarify this assessment.

Information provided includes:

• too low (n = 18): other research; data provided by the National Treatment Agency (NTA); own experience

• too high (N = 7): errors in model figures; too high relative to numbers on treatment; estimates derived from the British Crime Survey (BCS)

• don't know/other (N = 8): can't comment; no data; not in post in 2001

The basis of estimates included: research study (e.g. capture-recapture, economic modelling); need assessments; extrapolation from number in treatment, number of arrests; extrapolation from the British Crime Survey.

### Further analysis of Injecting Drug Use (IDU)

Of the 69 DATs who said their IDU estimates were either too low, too high or don't know, 37 (53%) provided some free text information to clarify this assessment.

Information provided included:

• too low (n = 27): figures suggest service capacity rather than actual figure; perception, based on proportion of problematic users who are injectors; model figures wrong

• too high (N = 2): local research; perception that smoking more common

• don't know/other (N = 8): can't comment; no data; not in post in 2001

**Figure 1:**
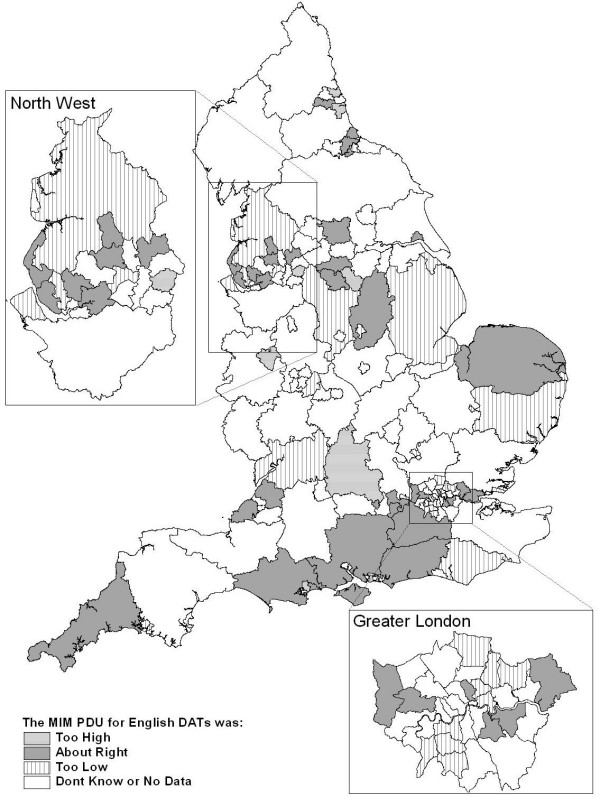
Drug Action Teams' perception of the Multiple Indicator Method (MIM) estimate of problematic drug use (PDU).

Figure 1 shows the geographical distribution of DATs' perception of their MIM estimate for problematic drug use.

### Comparison of DATs own PDU estimates and the MIM PDU estimates

**Figure 2:**
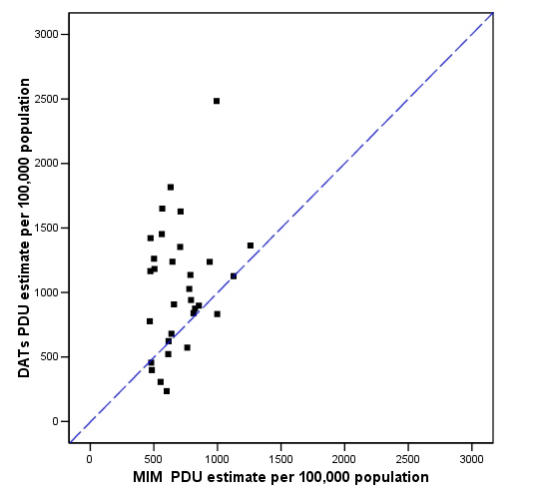
Drug Action Teams' own PDU estimates (Y axis) and the Multiple Indicator Method-Problematic Drug Use (MIM PDU) estimate (X axis).

Figure 2 shows that where DATs had PDU estimates of their own, they tended to be higher than their corresponding MIM estimates. However, it should be noted that the basis of these estimates require careful evaluation (e.g. methodology, target population, time period). In many cases DATs' own estimates may not be directly comparable with the MIM estimates.

6 of the 42 DATs (14%) who thought their MIM estimate were either 'too low' or 'too high' compared to the MIM estimate, actually had estimates within 10% of the MIM estimate.

### Comparison of DATs own IDU estimates and the MIM IDU estimates

As with PDU, where DATs had their own IDU estimates (N = 38) they tended to be higher than the MIM estimates. Two DATs who thought that their MIM IDU estimate were either 'too high' or 'too low', provided an estimate of their own that was within 10% of the MIM estimate.

### Evaluation of drug indicators

There were many reasons why DATs' own estimates/figures diverged from the MIM indicators. For example some DATs thought that indicator S31-refers to all people currently in treatment. This would account for the fact that for this indicator, 35 DATs thought the MIM figure was too low. In fact the MIM indicator refers to people entering treatment [Number of individuals aged 18+ with addresses in the DAT area recorded by RDMD as having entered treatment during 2000/2001].

In contrast, the majority of DATs thought that the MIM possession and supply figures were too high. One DAT suggested that their figure was a concatenation of 3 years figures. For five of the indicators, more DATs (who expressed an opinion) though that the indicators were "about right" as opposed too low/high.

Some DATs thought the methadone prescription figures were too low. These DATs had access to data on methadone prescriptions, but our data refer only to prescriptions issued in primary care. Where prescriptions are issued in secondary care, they would not be included in our data source.

These ambiguities could possibly have been reduced had the questionnaire provided more detail. However, there was concern that providing this information would have inhibited some DATs from responding. DATs were told that the further information on the indicators was contained in the full report which could be accessed online [18].

### Further comments on the estimates and indicators

44 DATs provided comments on the form. These covered a wide range of themes. Some DATs were anxious that estimates were wrong because of wrong input data. As explained above, this might have been due to misinterpretation of which data were actually used in the estimation process. Some DATs were concern that definition of PDU did not cover PDU as perceived in DAT area. This is a fair comment, but needs to be seen in the context of providing uniform estimates across all English DATs. Some DATs felt that it was difficult to comment on the situation in 2001 or that the relevancy of 2001 estimates was questionable. Finally some DATs were appreciative of the estimates and wished to be more directly involved in future estimation work.

## Discussion

### Main findings of this study

The MIM pilot study provided all DATs with estimates of the prevalence of problem drug use and injecting drug use in their area. Among responding DATs (N = 90, response rate = 60%), 33% thought the PDU estimates were "about right", while 24% were unable to make an assessment. A further 6 (7%) DATs who rated the MIM estimates as being too low/high provided their own estimate which was actually within 10% of the MIM estimate. By combining the three categories: "about right", "don't knows" and "within 10%", 64% thought the PDU estimates were about right or did not dispute them [The 'didn't know' DATs have been included on the basis that they had no local knowledge with which to make a comparison and therefore the MIM estimate is an improvement on no knowledge.]

Among responding DATs (n = 90, response rate = 60%), 23% thought the IDU estimates were "about right", while 27% were unable to make an assessment. A further 2 (2%) DATs who rated the MIM estimates as being too low/high provided their own estimate which was actually within 10% of the MIM estimate. Thus, by combining "about right", "don't knows" and "within 10%", 52% of DATs have potentially acceptable IDU estimates.

Of the 31 DATs who had their own PDU estimates, 24 of these were higher than the MIM estimate while 7 were lower. Of the 20 DATs who had their own IDU estimates, 17 of these were higher than the MIM estimate while 3 were lower. As noted above these estimates may not be comparable with the MIM estimates. Nevertheless, the reasons for so many DATs having higher estimates requires further investigation.

### What this study adds

This is the first study to determine the validity of estimates of problematic drug use in the UK. 64%/52% of the DATs thought the PDU/IDU estimates were about right or did not dispute them. However, these figures are lower if the "don't knows" are excluded (40% and 25% respectively), and further work is needed to determine the reasons for this discrepancy.

### Limitations of the study

Many comments were received on the validity and reliability of the indicators. For example, most DATs thought the indicator on the number in treatment was too low, that the possession and supply indicators were too high, that arrest referral and drug related deaths were about right, and did not know about methadone referrals or deprivation. Clearly, if there is heterogeneity in the completeness of accuracy of indicators this will add bias to the MIM estimates, and this may be a potential explanation for why the MIM estimates were considered too low or too high. For example, the MIM study indicated that in one particular DAT there were no IDUs, while the DAT itself reported in excess of 100 needle exchange attendances.

## Conclusion

Given that public health expenditure on illicit drug use is based on estimates of the scale of the problem, validation should be an inherent feature of prevalence estimation studies. Recently, researchers have estimated problematic drug use for all DATs in England using capture-recapture for all DATs (except for a few where sufficient data were not available). The definition of problematic drug use was use of opiates and/or the use of crack cocaine which is narrower than the definition used in the MIM study (which also included benzodiazepines). The estimate for the median number of problematic drug users in England for 2004/5 was 327,466 [19]. This figure represents a 14% increase on the 2001 MIM study. However, between 2001 and 2005 the rate of self-reported class A drug use in the last month among people aged 16–59 in the population dropped by 14% [20] and the number of drug related deaths declined by 10% [21]. These figures suggest that problematic drug use in England declined between 2001 and 2005.

These findings have implications for future studies as there are two approaches to prevalence estimation for English DATs. The first approach would involve conducting capture-recapture studies for all DATs. The second approach would involve conducting capture-recapture studies in a small number of DATs and using the MIM to estimate prevalence for remaining DATs. The results of this paper highlight the need to consider criterion and face validity when evaluating estimates of the number of drug users.

## Competing interests

The author(s) declare that they have no competing interests.

## Authors' contributions

The study was conceived by MF. MF, HH and MH developed the study protocol. The data were analysed by MF and HH. The manuscript was written by MF, HH and MH. All authors read and approved the final manuscript.

## Pre-publication history

The pre-publication history for this paper can be accessed here:


